# How does the dengue vector mosquito *Aedes albopictus* respond to global warming?

**DOI:** 10.1186/s13071-017-2071-2

**Published:** 2017-03-11

**Authors:** Pengfei Jia, Xiang Chen, Jin Chen, Liang Lu, Qiyong Liu, Xiaoyue Tan

**Affiliations:** 1 College of Global Change and Earth System Science, Beijing Normal University, Beijing, 100875 China; 2 State Key Laboratory of Earth Surface Processes and Resource Ecology, Beijing Normal University, Beijing, 100875 China; 3 Department of Emergency Management, Arkansas Tech University, Russellville, 72801 AR USA; 4 National Institute for Communicable Disease Control and Prevention, China CDC, Beijing, 102206 China

**Keywords:** *Aedes albopictus*, Mosquito, Global warming, Seasonality, Mechanistic population modeling, Thermal reaction norms

## Abstract

**Background:**

Global warming has a marked influence on the life cycle of epidemic vectors as well as their interactions with human beings. The *Aedes albopictus* mosquito as the vector of dengue fever surged exponentially in the last decade, raising ecological and epistemological concerns of how climate change altered its growth rate and population dynamics. As the global warming pattern is considerably uneven across four seasons, with a confirmed stronger effect in winter, an emerging need arises as to exploring how the seasonal warming effects influence the annual development of *Ae. albopictus*.

**Methods:**

The model consolidates a 35-year climate dataset and designs fifteen warming patterns that increase the temperature of selected seasons. Based on a recently developed mechanistic population model of *Ae. albopictus*, the model simulates the thermal reaction of blood-fed adults by systematically increasing the temperature from 0.5 to 5 °C at an interval of 0.5 °C in each warming pattern.

**Results:**

The results show the warming effects are different across seasons. The warming effects in spring and winter facilitate the development of the species by shortening the diapause period. The warming effect in summer is primarily negative by inhibiting mosquito development. The warming effect in autumn is considerably mixed. However, these warming effects cannot carry over to the following year, possibly due to the fact that under the extreme weather in winter the mosquito fully ceases from development and survives in terms of diapause eggs.

**Conclusions:**

As the historical pattern of global warming manifests seasonal fluctuations, this study provides corroborating and previously ignored evidence of how such seasonality affects the mosquito development. Understanding this short-term temperature-driven mechanism as one chain of the transmission events is critical to refining the thermal reaction norms of the epidemic vector under global warming as well as developing effective mosquito prevention and control strategies.

**Electronic supplementary material:**

The online version of this article (doi:10.1186/s13071-017-2071-2) contains supplementary material, which is available to authorized users.

## Background

How global warming potentially influences vector-borne diseases has prompted great concerns from both the general public and ecological scientists [1, 2]. Dengue fever, known as the most prevalent mosquito-borne epidemic, generates 50–100 million cases per year and is constantly growing with the expansion of urban residence, increased air travel and the growth of global population [2–5]. In addition to human-induced factors, the observed global warming trend plays a potential role in the generation and transmission of the disease [6]. The complex interplay between global warming and dengue viruses has been a subject of debate yet to be fully understood; one critical component in the long chain of transmission events is the altered population dynamics of the vector, *Aedes albopictus*, commonly known as the Asian Tiger mosquito [7–10]. Understanding how *Ae. albopictus* responds to global warming underscores the need to develop effective strategies to mitigate and control dengue and other mosquito-borne epidemics.

*Ae. albopictus* is native to the humid tropics of Southeast Asia [11]. With an increasing pace of globalization, the mosquito expanded its habitat to other continental regions, including East Asia, Europe, Africa, the Middle East and the Americas [6, 9, 11]. With an observed warmer global pattern in the last several decades, *Ae. albopictus* has geographically shifted from its original habitats [12, 13]. Specifically, the presence of the mosquito surged in certain high-latitude temperate areas, such as central north-western European and Balkan countries [14, 15]. The emergence was attributed to the rising local temperature, giving rise to more conductive habitats and lengthened activity periods that allowed for an improved rate of success in overwintering [14]. In addition, field observations confirmed a decline in the density of the species in areas with a recorded dry and warm summer, such as southern Spain and Sardinia [14]. This decline was explained by the rising temperature rendering the previously favored habitats less suitable [14, 16]. These contrasting responses displayed a potential geographical shift due to changes in mosquito habitats and behaviors as a result of warmer temperatures. These empirical studies further provided evidence for predicting the regional sustainability of the mosquito and controlling their infestation in areas of prevalence.

A step forward to accommodate these observations is to identify the climatic conditions that affect *Ae. albopictus* growth. In most world regions, temperature plays a dominating role in the development of the species, along with precipitation and photoperiod [17]. There has been a long tradition in biological research on the relationship between climatic factors and population dynamics of *Ae. albopictus*, using controlled laboratory experiments [18–20]. It is generally accepted that moderate-to-high temperatures expedite selected stages of the development, while shortening the lifespan of adult mosquitoes [21, 22]. These structured relationships have been incorporated in temperature-driven empirical models to demarcate areas suitable for mosquito development under projected future warming scenarios [14].

One element missing in the discussion is how global warming affects the mosquitoes’ life cycle in terms of different growth stages. This issue is introduced by two existing gaps in the research on the climate-driven nature of the *Ae. albopictus* population. First, although many studies are able to identify the favorable conditions for development, the contingencies between growth stages are often overlooked. The complete life cycle of the *Ae. albopictus* is a chain of events differing in morphology and natural habitats. Existing studies using controlled experiments and statistical modeling are invariably centered on limited growth stages (e.g. larvae, pupae), while the intrinsic connections between stages are ignored. Secondly, although a wide discussion arises regarding the projection of climatic suitability for the growth of mosquito in the context of global warming [13, 14, 16, 20], the seasonality of development (i.e. the population variance on a seasonal basis) rendered by warming effects has received very little attention to date. This issue is of great importance as the global warming pattern is considerably uneven across four seasons, with the greatest warming effect at high-latitudes in winter [23, 24]. Thus, an emergent need is to develop a rigorous mathematical model that illuminates the seasonal and stage-specific relationship and thus provides evidence for excavating how the mosquito responds to the heterogeneity of global warming.

Based on a recently proposed mechanistic population model [25], this study examines the theoretical thermal reaction of *Ae. albopictus* adults over four seasons of a year. By incorporating a 35-year historical dataset into the model, the study identifies critical metrics about the mosquito development. This study aims to provide a tentative answer to the effects of seasonality on *Ae. albopictus*, while shedding light on the assessment of the epidemic vector’s sustainability in a regional context.

## Methods

### Study area and data

The case study was conducted in Guangzhou, China (23°17’N, 113°23’E). Guangzhou, a transportation port located in south-western China, is the third largest city in the country. Guangzhou is under a typical subtropical climate with a humid, hot summer and a mild winter. The peculiar climate has created favorable conditions for mosquitoes to hatch and develop, giving rise to widespread cases of mosquito-borne diseases. The number of dengue cases in Guangzhou over the first 11 months of 2014 was 37,305, accounting for 70.75% of the cumulative cases since 1978 [26]. The sudden outbreak of dengue drew the public attention of its peridomestic vector to a heightened extent [27]. In a previous study, we established a climate-driven population model of *Ae. albopictus* [25]. Built upon this model, this study attempts to answer a broader question of how the mosquito responds to the temperature rise in the last four decades. To achieve this goal, we collected a 35-year climatic dataset based on one observation station in Guangzhou. The datasets included daily mean temperature and daily accumulative precipitation over 1980–2014 from the China Meteorological Data Sharing Service System [28]. Data of photoperiod were derived from the National Oceanic and Atmospheric Administration [29] with respect to the geographical coordinates of Guangzhou. These datasets were incorporated into the model to simulate the theoretical thermal reaction of *Ae. albopictus* under different warming scenarios.

### Mechanistic population model of *Ae. albopictus*

The mechanistic population model is different from the traditional statistical model, in that it formulates the successive development of the species in a bottom-up approach [25, 30]. In a mechanistic population model, an individual behavior (e.g. egg hatching) can be described by a mathematical equation (invariably in the form of a differential equation). By combining equations with each representing a single but related behavior, a mechanistic model is able to demonstrate and explain how the dependent variables affect the outcome at each phase of the development [30–32].

The model employed in the study is termed the mechanistic population model of *Ae. albopictus* with diapause (MPAD). The model was developed by Jia et al. [25] to characterize the population dynamics of *Ae. albopictus* at different growth stages of its life cycle. The mosquito’s life cycle can be broadly dichotomized into the aquatic period and the aerial period with different compartments [33]. The MPAD model establishes a seven-stage life cycle, including eggs (diapause *E*_0_ and non-diapause *E*_dia_), larvae (*L*), pupae (*P*), emerging adults (*A*_em_), blood-fed adults (*A*_b_), gestating adults (*A*_g_) and ovipositing adults (*A*_r_). These stages are formulated by a set of seven differential equations to describe the successive development process of *Ae. albopictus*, as shown in Eq. (1). The parameters included in the equation are given in Table 1. In Eq. (1), the dependent variable of each differential equation denotes the daily variation of population abundance at a specific life stage. The climate-independent parameters in Table 1 are primarily driven by three climatic factors: daily mean temperature (*T*), daily accumulative precipitation (*PP*) and daily photoperiod (*SD*). Table 2 summarizes the relationship between these parameters and the climatic factors.

**Table 1:** Notation of the MPAD model parameters

Climate-dependent parameter	
*f*_E_	Non-diapause egg hatching rate (day^-1^)
*f*_dia_	Diapause egg hatching rate (day^-1^)
*f*_L_	Larval development rate (day^-1^)
*f*_P_	Pupal development rate (day^-1^)
*m*_L_	Larval mortality rate (day^-1^)
*m*_P_	Pupal mortality rate (day^-1^)
*m*_A_	Adult mortality rate (day^-1^)
*β*	Oviposition rate by each female (day^-1^)
*f*_Ag_	Gestating adult development rate (day^-1^)
*k*_L_	Environmental carrying capacity for larvae (ha^-1^)
*k*_P_	Environmental carrying capacity for pupae (ha^-1^)
*z*_1_	Binary function for diapause eggs oviposited
*z*_*2*_	Binary function for diapause egg hatching
*z*_dia_	Binary function for adult activity during the diapause
Climate-independent parameter	
*m*_*E*_	Non-diapause egg mortality rate (day^-1^)
*m*_*dia*_	Diapause egg mortality rate (day^-1^)
*σ*	Percentage of females at emergence stage
*μ*_*em*_	Emerging adult mortality rate (day^-1^)
*μ*_*r*_	Adult mortality rate related to seeking behavior (day^-1^)
*γ*_*Aem*_	Emerging adult development rate (day^-1^)
*γ*_*Ab*_	Blooding adult development rate (day^-1^)
*γ*_*Ao*_	Ovipositing adult development rate (day^-1^)

**Table 2:** Relationship between climate-dependent parameters and climatic factors

Parameter	Equation
*f*_E_	${f}_{\mathrm{E}}(T)=0.5070 \exp \left[-{\left(\frac{T-30.85}{12.82}\right)}^2\right]$
*f*_dia_	${f}_{\mathrm{dia}}(T)=0.1*0.5070 \exp \left[-{\left(\frac{T-30.85}{12.82}\right)}^2\right]$
*f*_L_	${f}_{\mathrm{L}}(T)=0.1727 \exp \left[-{\left(\frac{T-28.40}{10.20}\right)}^2\right]$
*f*_P_	${f}_{\mathrm{P}}(T)=0.6020 \exp \left[-{\left(\frac{T-34.29}{15.07}\right)}^2\right]$
*m*_L_	${m}_{\mathrm{L}}(T)= min\left\{1,\frac{1}{\left|-0.1305{T}^2+3.868 T+30.83\right|}\right\}$
*m*_P_	${m}_{\mathrm{P}}(T)= min\left\{1,\frac{1}{\left|-0.1502{T}^2+5.057 T+3.517\right|}\right\}$
*m*_A_	${m}_{\mathrm{A}}(T)= min\left\{1,\frac{1}{\left|-0.1921{T}^2+8.147 T-22.98\right|}\right\}$
*β*	*β*(*T*) = *max*{0, − 0.0162*T* ^2^ + 1.289*T* − 15.837}
*f*_Ag_	${f}_{\mathrm{Ag}}(T)= max\left\{0,\frac{T-10}{77}\right\}$
*k*_L_	*k*_*L*_(*PP*_*norm*_) = *κ*_*L*_(1 + *PP*_*norm*_)
*k*_P_	*k*_*P*_(*PP*_*norm*_) = *κ*_*P*_(1 + *PP*_*norm*_)
*z*_1_	z_1_(*T*_*ave*_, *SD*_*ave*_) = $$ \left\{\begin{array}{c}\hfill 1,\ {T}_{ave}(t)<21{}^{\circ}C\ and\ S{D}_{ave}(t)<13.5 h,\ {t}_{eggBegin}< t<{t}_{diaBegin}\hfill \\ {}\hfill 0,\kern0.5em otherwise\hfill \end{array}\right. $$
*z*_*2*_	z_2_(*T*_*ave*_, *SD*_*ave*_) = $$ \left\{\begin{array}{c}\hfill 1,\ {T}_{ave}(t)>10.5{}^{\circ}C\ and\ S{D}_{ave}(t)>10.25 h,\ {t}_{diaEnd}< t<{t}_{eggEnd}\hfill \\ {}\hfill 0,\kern0.5em otherwise\hfill \end{array}\right. $$
*z*_dia_	z_2_(*T*_*ave*_, *SD*_*ave*_) = $$ \left\{\begin{array}{c}\hfill 1,\ {T}_{ave}(t)<9.5{}^{\circ}C,\ t>{t}_{diaBegin}\ or\ t<{t}_{diaEnd}\hfill \\ {}\hfill 0,\kern0.5em otherwise\hfill \end{array}\right. $$

*Abbreviations*: *T* daily mean temperature, *PP*_*norm*_ rainfall over a 2-week period, normalized between 0 and 1 [32], *T*_*ave*_ 7-day averaged daily mean temperature, *SD*_*ave*_ 7-day averaged daily sunlight hour, *t*_*eggBegin*_ the time when diapause eggs emerge, *t*_*eggEnd*_ the time when diapause eggs finish hatching, *t*_*diaBegin*_ the time when diapause period begins, *t*_*diaEnd*_ the time when diapause period ends

A major contribution of the model is the consideration of diapause, referred to as the state of the mosquito eggs being dormant and unable to hatch under extreme weather and desiccation [34, 35]. The model formulates egg diapause with a binary variable (*z*_dia_) and related growth parameters, such as mortality rate (*m*_dia_) and development rate (*f*_dia_). As the adult mosquitoes become naturally eradicated when the temperature drops under 9.5 °C [36], the model assumes that this is the thermal threshold of diapause. Thus the model identifies the day of year (DOY) when the diapause begins and ends, eventually establishing the temperature-driven mechanism for egg/larva/pupa abundance based on their mortality rate and development rate. Through validation by the container index (CI) data collected from two Chinese cities over two respective 5-year periods (i.e. Guangzhou over 2007–2011 and Shanghai over 2009–2013), the model has achieved a relatively good fitting (i.e. correlation coefficient *r* = 0.84 for Guangzhou and *r* = 0.90 for Shanghai) [25]. The code of the model is provided in the reference [37].

1 $$ \left\{\begin{array}{l}{\overset{.}{E}}_0=\left(1-{z}_1\right)\;\beta\;{A}_0-\left({m}_E+{f}_E\right)\;{E}_0\\ {}{\overset{.}{E}}_{dia}={z}_1\;\beta\;{A}_0-\left({m}_{dia}+{z}_2\;{f}_{dia}\right)\;{E}_{dia}\\ {}\overset{.}{L}=\left({f}_E\;{E}_0+{z}_2\;{f}_{dia}\;{E}_{dia}\right)-\left[{m}_L\;\left(1+ L/{k}_L\right)+{f}_L\right]\; L\\ {}\overset{.}{P}={f}_L\; L-\left({m}_P+{f}_P\right)\; P\\ {}{\overset{.}{A}}_{e m}={f}_P\;\sigma\;{e}^{-{\mu}_{e m}\;\left(1+ P/{k}_P\right)}\; P-\left({m}_A+{z}_{dia}\;{\gamma}_{A em}\right)\;{A}_{e m}\\ {}{\overset{.}{A}}_b={z}_{dia}\;\left({\gamma}_{A em}\;{A}_{e m}+{\gamma}_{A o}\;{A}_o\right)-\left({m}_A+{\mu}_r+{z}_{dia}\;{\gamma}_{A b}\right)\;{A}_b\\ {}{\overset{.}{A}}_r={z}_{dia}\;{\gamma}_{A b}\;{A}_b-\left({m}_A+{f}_{A g}\right)\;{A}_g\\ {}{\overset{.}{A}}_o={f}_{A g}\;{A}_g-\left({m}_A+{\mu}_r+{z}_{dia}\;{\gamma}_{A o}\right)\;{A}_o\\ {} E={E}_0+{E}_{dia}\end{array}\right. $$

### Temperature data preprocessing

**Fig. 1:**
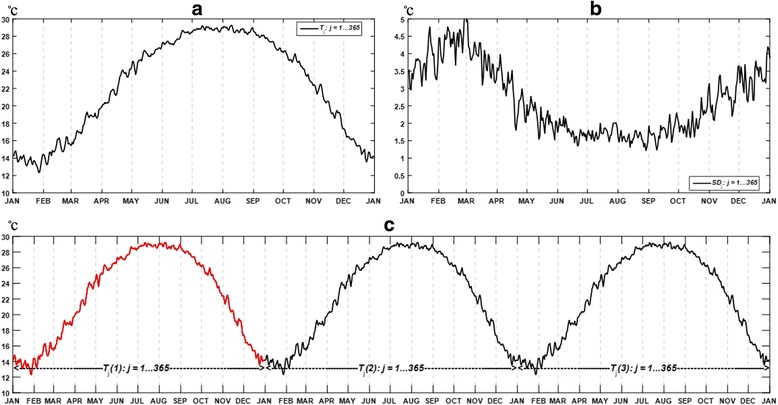
**a** Average daily mean temperature over 1980–2014. **b** Average standard deviation of the daily mean temperature over 1980–2014. **c** Expanded 3-year temperature based on the average daily mean temperature over 1980–2014, where Year 2 and Year 3 are assumed the same as the first year

As the long-term climate manifests a high degree of variability, we consolidated the 35-year data into a single year to represent the typical annual climatic conditions. Equation (2) gives the formula for the average daily temperature (*T*_j_) on the *j*th day of the consolidated year, along with its graphic shown in Fig. 1a. We also derived the average standard deviation of the daily mean temperature in the same manner, as shown in Fig. 1b. The standard deviation of the temperature is used to support the degree of temperature increase in the follow-up simulations.

2 $$ {T}_j=\frac{1}{35}\;{\displaystyle \sum_{i=1}^{35}{T}_j^i, j=1,\dots, 365} $$

Using the temperature of a single year does not suffice to test the warming effect, as in simulation models the result is largely influenced by the initial configuration of temperatures [25, 30, 32]. Thus, we extrapolated the temperatures for Year 2 and 3 based on the temperature in Year 1, indicated by *T*_j_(*k*) (*k* = 1…3, *j* = 1…365) (Fig. 1c). The daily accumulative precipitation (*PP*_j_) and daily photoperiod (*SD*_j_) on the *j*th day of the year as required model parameters were processed in the same manner, as shown in Eqs. (3) and (4).

3 $$ P{P}_j=\frac{1}{35}\;{\displaystyle \sum_{i=1}^{35} P{P}_j^i, j=1,\dots, 365} $$

4 $$ S{D}_j=\frac{1}{35}\;{\displaystyle \sum_{i=1}^{35} S{D}_j^i, j=1,\dots, 365} $$

### Design of warming patterns

Research on global warming has found the warming effect is uneven across geographical scales as well as over different seasons, where the winter season at high latitudes manifests the highest level of increase [24]. Existing studies on the projected climatic suitability of mosquitoes are invariably focused on the long-term, regional impact, whereas the short-term, temporal variance has been less explored [14, 16, 20, 38]. Mosquitoes as a typical ectotherm are highly susceptible to ambient temperatures; and therefore, their physiological responses and strategic adaptations to regional warming are more complex than a simple linear pattern [39]. Specifically, seasonality is a significant indicator of the warming tolerance as well as the thermal limits of ectotherms, constraining the development, reproduction, dormancy and migration of the population [39]. To explore the intricacy of seasonal responses, we systematically adjusted the temperatures of selected seasons in order to identify the impact on the second year (Year 2) and the third year (Year 3) populations. According to Fig. 1b, the local temperature fluctuated within a 5 °C range over the past 35 years. We then considered the temperature increase (∆*T*) to be a variable ranging from 0.5 to 5 °C at an interval of 0.5 °C, which is a slightly broader range than an estimated global temperature rise by 1.8 to 4 °C in the decade to come [40].

To explore how seasonality affects the development of *Ae. albopictus*, we strategically designed four warming categories, as given below:

- All-year warming (YW): increasing the temperature by ∆*T* for all four seasons.
- Single-season warming (SW1): increasing the temperature by ∆*T* for a single season.
- Two-season warming (SW2): increasing the temperature by ∆*T* for two selected seasons.
- Three-season warming (SW3): increasing the temperature by ∆*T* for three selected seasons.

By considering different seasonal combinations under each category, we generated a total of fifteen seasonal warming patterns, representing the complex nature of global warming. The selected warming months to be tested and their temporal divides in DOY are given in Table 3, where the temperature adjustments are mostly in Year 2.

**Table 3:** Warming patterns and their included warming months

Warming pattern	Selected warming months	Adjusted temperature (*j* = DOY)
YW	January-December, 2^nd^ year	*T*_j_(2) + ∆*T* ^a^, *j* = 1–365
SW1-Spr	March-May, 2^nd^ year	*T*_j_(2) + ∆*T*, *j* = 60–151
SW1-Sum	June-August, 2^nd^ year	*T*_j_(2) + ∆*T*, *j* = 152–243
SW1-Aut	September-November, 2^nd^ year	*T*_j_(2) + ∆*T*, *j* = 244–304
SW1-Win	December, 2^nd^ year; January-February, 3^rd^ year	*T*_j_(2) + ∆*T*, *j* = 305–365 *T*_j_(3) + ∆*T*, *j* = 1–59
SW2-Spr-Sum	March-August, 2^nd^ year	*T*_j_(2) + ∆*T*, *j* = 60–243
SW2-Spr-Aut	March-May; September-November, 2^nd^ year	*T*_j_(2) + ∆*T*, *j* = 60–151 *T*_j_(2) + ∆*T*, *j* = 244–304
SW2-Sum-Aut	June-November, 2^nd^ year	*T*_j_(2) + ∆*T*, *j* = 152–304
SW2-Sum-Win	June-August and December, 2^nd^ year; January-February, 3^rd^ year	*T*_j_(2) + ∆*T*, *j* = 152–243 *T*_j_(2) + ∆*T*, *j* = 305–365 *T*_j_(3) + ∆*T*, *j* = 1–59
SW2-Aut-Win	September-December, 2^nd^ year; January-February, 3^rd^ year	*T*_j_(2) + ∆*T*, *j* = 244–365 *T*_j_(3) + ∆*T*, *j* = 1–59
SW2-Win-Spr	December, 2^nd^ year; January-May, 3^rd^ year	*T*_j_(2) + ∆*T*, *j* = 305–365 *T*_j_(3) + ∆*T*, *j* = 1–151
SW3-Sum-Aut-Win	June-December, 2^nd^ year; January-February, 3^rd^ year	*T*_j_(2) + ∆*T*, *j* = 152–365 *T*_j_(3) + ∆*T*, *j* = 1–59
SW3-Aut-Win-Spr	September-December, 2^nd^ year; January-May, 3^rd^ year	*T*_j_(2) + ∆*T*, *j* = 244–365 *T*_j_(3) + ∆*T*, *j* = 1–151
SW3-Win-Spr-Sum	December, 1^st^ year; January-August, 2^nd^ year	*T*_j_(1) + ∆*T*, *j* = 305–365 *T*_j_(2) + ∆*T*, *j* = 1–243
SW3-Spr-Sum-Aut	March-November, 2^nd^ year	*T*_j_(2) + ∆*T*, *j* = 60–304

^a^∆*T* is the temperature increase by 0.5–5 °C at an interval of 0.5 °C

*Abbreviations*: *Aut* autumn, *Spr* spring, *Sum* summer, *Win* winter

By implementing the adjusted temperatures into the MPAD model, results regarding the *Ae. albopictus* population dynamics can be simulated and derived. The paper mainly explores the phase of blood-fed adults, as it is the most critical step for the mosquito to complete a gonotrophic cycle and transition into a viral vector [41]. We derived the following variables to describe the nature and growth of blood-fed adults:

- Blood-fed adult population *A*_b_ (*j*, ∆*T*): defined as the daily population of blood-fed adults when the temperature increases by ∆*T* (1 ≤ *j* ≤ 365, ∆*T* = 0:0.5:5 °C).
- Peak value *P*_Ab_ (∆*T*): defined as the maximum daily population in a life cycle when the temperature increases by ∆*T*.
- Peak time *t*_p_ (∆*T*): defined as the DOY when *P*_Ab_ appears.
- Emergence time *t*_e_ (∆*T*): defined as the DOY when the ratio of *A*_b_ to *P*_Ab_ first exceeds 10% as the temperature increases by ∆*T*.
- Exit time *t*_d_ (∆*T*): defined as the DOY when the ratio of *A*_b_ to *P*_Ab_ first drops below 10% as the temperature increases by ∆*T*.
- Percentage change of peak value *V*_peak_ (∆*T*): defined as the change of peak value in relation to the unadjusted peak value as the temperature increases by ∆*T*, as shown in Eq. (5).
  5 $$ {V}_{\mathrm{peak}}={V}_{\mathrm{peak}}\;\left(\varDelta T\right)=\frac{P_{\mathrm{Ab}}\;\left(\varDelta T\right)-{P}_{\mathrm{Ab}}\;(0)}{P_{\mathrm{Ab}}\;(0)}. $$
- Percentage change of population abundance *V*_amount_ (∆*T*): defined as the change of blood-fed population abundance in relation to the unadjusted blood-fed adult population, as shown in Eq. (6).
  6 $$ {V}_{\mathrm{amount}}={V}_{\mathrm{amount}}\;\left(\varDelta T\right)=\frac{{\displaystyle \sum_{j={t}_{\mathrm{e}}}^{t_{\mathrm{d}}}{A}_{\mathrm{b}}\;\left( j,\varDelta T\right)}-{\displaystyle \sum_{j={t}_{\mathrm{e}}}^{t_{\mathrm{d}}}{A}_{\mathrm{b}}\;\left( j,0\right)}}{{\displaystyle \sum_{j={t}_{\mathrm{e}}}^{t_{\mathrm{d}}}{A}_{\mathrm{b}}\;\left( j,0\right)}}. $$

## Results

**Fig. 2:**
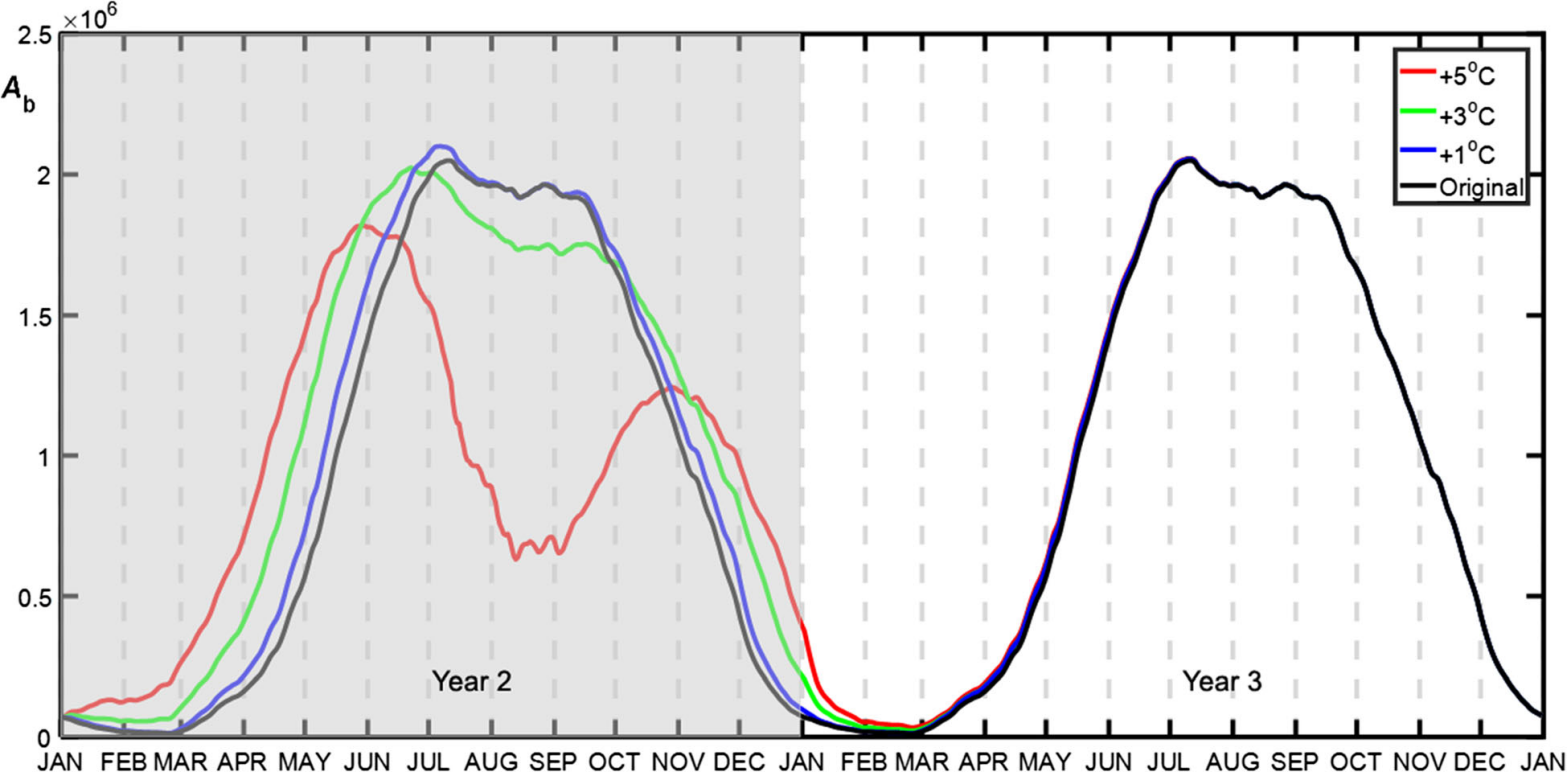
Blood-fed adult population (*A*_b_) by implementing the all-year warming pattern with ∆*T* = 1, 3 and 5 °C, respectively. The *shaded* area represents the warming period

**Fig. 3:**
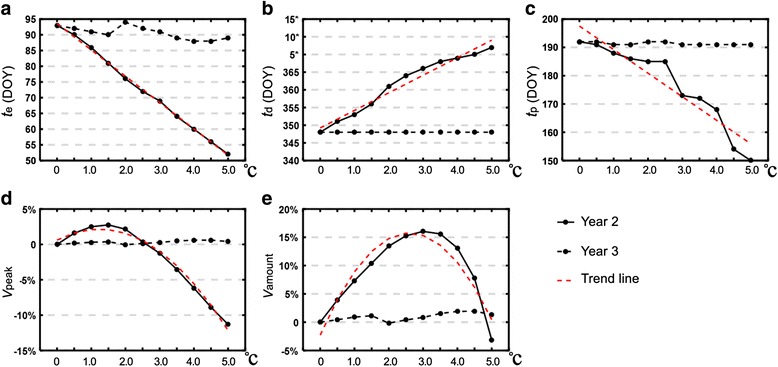
Attributes of blood-fed adult population in Year 2 and Year 3 with respect to *ΔT* in the YW pattern. The *shaded* area is the warming period. The attributes include: **a** emergence time (*t*_e_) in DOY; **b** exit time (*t*_d_) in DOY; **c** peak time (*t*_p_) in DOY; **d** percentage change of peak value (*V*_peak_); and **e** percentage change of population abundance (*V*_amount_)

**Fig. 4:**
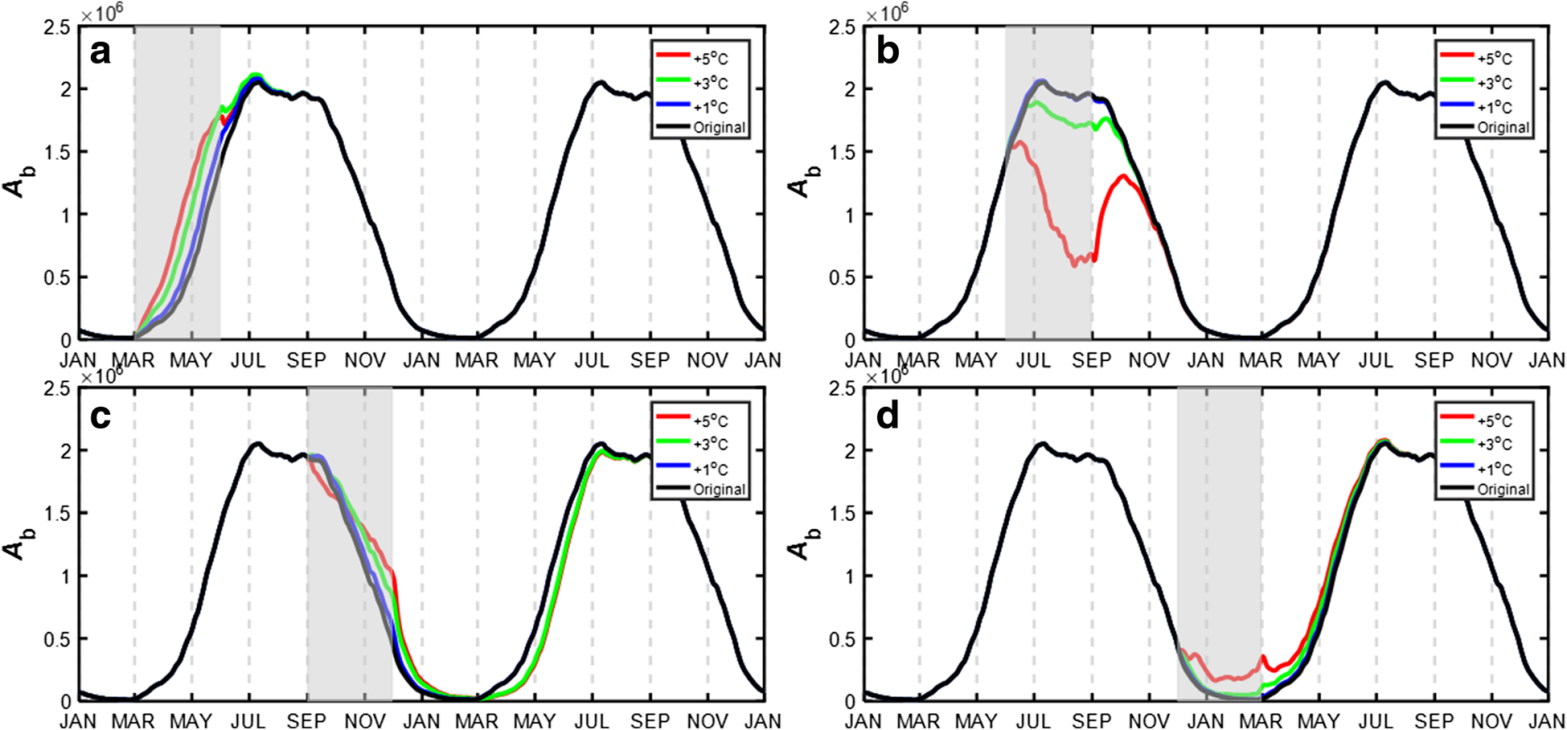
Blood-fed adult population (*A*_b_) by implementing the single-season warming pattern of: **a** SW1-Spring; **b** SW1-Summer; **c** SW1-Autumn; and **d** SW1-Winter with ∆*T* = 1, 3 and 5 °C, respectively. The *shaded* area represents the warming period

**Fig. 5:**
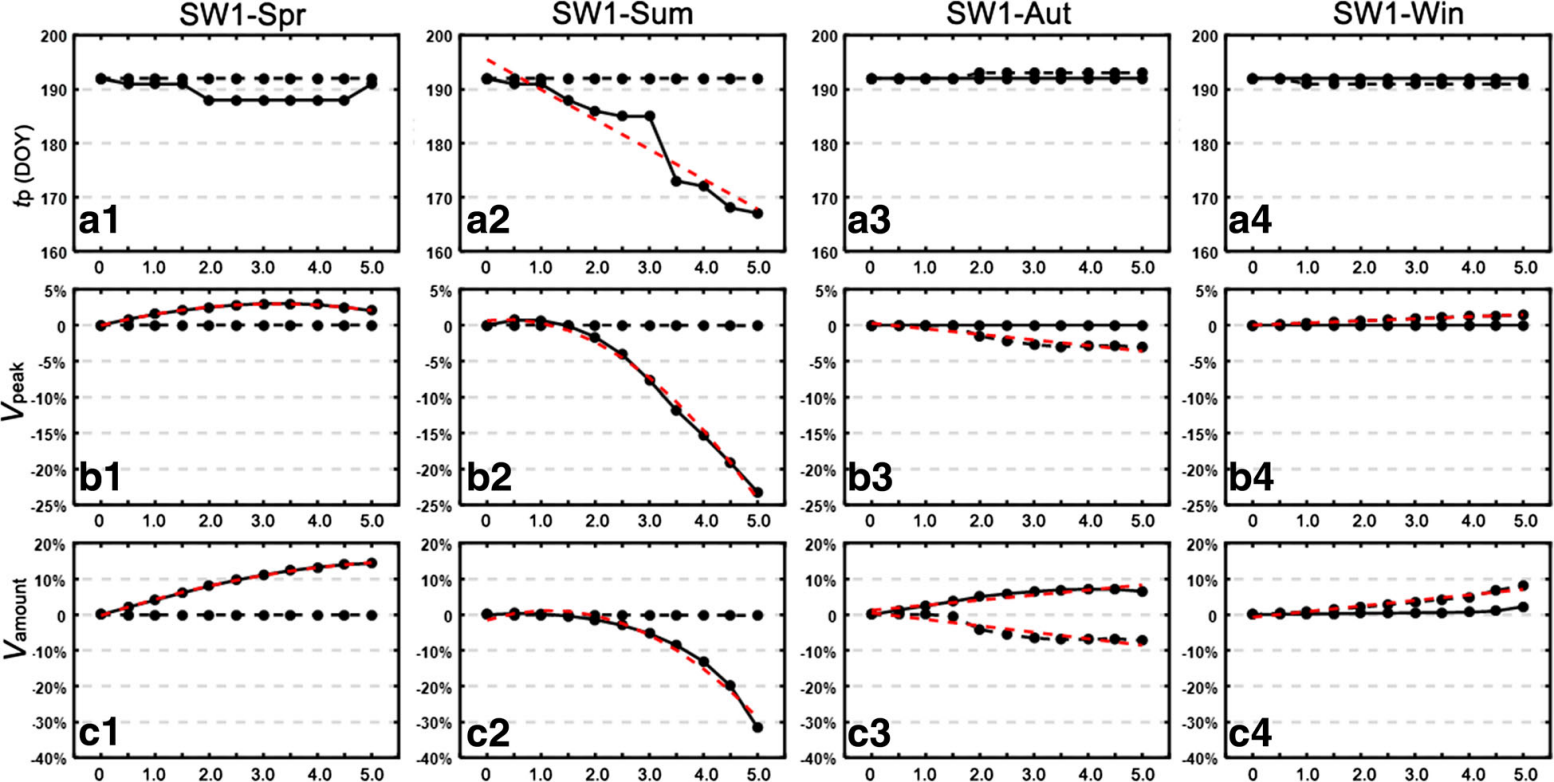
Attributes of blood-fed adult population in Year 2 and Year 3 with respect to ∆*T* in the four SW1 patterns. The *shaded* area represents the warming period. The attributes include: peak time (*t*_p_) in DOY, percentage change of peak value (*V*_peak_) and percentage change of population abundance (*V*_amount_)

By plugging each of the fifteen warming patterns into the MPAD model and adjusting the temperature by ∆*T* (0.5–5 °C), new population curves as a function of DOY were derived. Here we mainly explore the effects of one YW pattern (Figs. 2 and 3) and four SW1 warming patterns (Figs. 4 and 5), while the results for SW2 (Additional file 1: Figure S1) and SW3 (Additional file 1: Figure S2) patterns are given in Additional file 1. We also performed a regression analysis for each of the population variables (*t*_p_, *t*_e_, *t*_d_, *V*_peak_ and *V*_amount_) as a function of *ΔT* and achieved a relatively good model fitting. These results are given in Additional file 2.

### All-year warming (YW)

Figure 2 shows the population dynamics of the blood-fed adults before and after the temperature increases in Year 2. Compared to the original population curve (black line), the all-year warming scenario had very contrasting effects on the population abundance of Year 2: a minor increase by ∆*T* = 1 °C facilitated the development to a limited extent, where the peak population surfaced in July (DOY = 188); a drastic increase (e.g. 5 °C) reshaped the thermal reaction and caused a noticeable drop in late summer. However, the temperature increase in Year 2 did not have a cascading effect on Year 3 for the most part, whereas only the first 3 months of Year 3 were affected.

Figure 3 shows the five metrics of the blood-fed adults’ development cycle with respect to the degree of warming in Year 2. It can be seen from the diagram that the modulated temperature had a marked influence on the population dynamics of Year 2, whereas Year 3 was less affected. The temperature had an overarching effect on the development period by shifting it to an earlier emergence time *t*_e_ (Fig. 3a) and a later exit time *t*_e_ (Fig. 3b). The peak time *t*_p_ had a quasi-linear relationship with ∆*T*; and it was advanced by a maximum of 42 days at ∆*T* = 5 °C (Fig. 3c). A curvilinear relationship was identified between *∆T* and the percentage change of the peak value *V*_peak_ (Fig. 3d) as well as between ∆*T* and the percentage change of population abundance *V*_amount_ (Fig. 3e), where the peak of *V*_peak_ (2.8%) appeared at *ΔT* = 1.5 °C and that of *V*_amount_ (16.0%) at *ΔT* = 3.0 °C.

### Single-season warming (SW1)

Figure 4 shows the warming effect in a single season on the population abundance. The increased temperature facilitated the development in all seasons but summer, where the curve showed two peaks with different magnitudes (Fig. 4b). This pattern became most evident as the temperature rose by ∆*T* = 5.0 °C. The spring increase expedited the early growth of the population by advancing the emergence time by a maximum of 24 days at ∆*T* = 5.0 °C (Fig. 4a); the autumn increase slightly postponed the exit time by a maximum of 15 days at ∆*T* = 5.0 °C (Fig. 4c). In addition, the impact on Year 3 was generally very minor except autumn and winter: the autumn increase caused a slight drop of the population from March through August in Year 3 (Fig. 4c); and the winter increase facilitated the development from January through July in Year 3 (Fig. 4d).

Figure 5 shows three major indices regarding the population abundance. Again the effect was not pronounced in Year 3. The summer increase had a negative influence with a reduced peak value (*V*_peak_ down by 0–23.2%, Fig. 5b2) and a declined overall population abundance (*V*_amount_ down by 0–31.5%, Fig. 5c2). The spring increase and winter increase had a positive influence on the population growth (spring: *V*_peak_ up by 0–3.0%, Fig. 5b1, *V*_amount_ up by 0–14.4%, Fig. 5c1; winter: *V*_peak_ up by 0–2.2%, Fig. 5b4, *V*_amount_ up by 0–8.2%, Fig. 5c4), whereas the effect was relatively mixed for the autumn increase.

## Discussion

As an extension of the MPAD model, the paper provides a new perspective to examine how the mosquito *Ae. albopictus* responds to warming temperatures, a problem that raises both ecological and epistemological concerns. To accommodate former studies that established the thermal reaction norms using controlled experiments [42], this study is the first to explore the seasonality of warming effects on *Ae. albopictus* adults implemented in a mechanistic population model. One persisting issue in controlled experiments and field surveys is that monitoring mosquito adults is relatively difficult; and the larval/pupal surveillance may misrepresent the adult population abundance [43, 44]. To this end, the established MPAD model is able to simulate and elucidate the nature of the thermal reaction at each phase of the life cycle while providing corroborating evidence of how the seasonality affects the overall population abundance.

The historical pattern of global warming manifests temporal fluctuations, characterized by warmer winter temperatures at northern latitudes [23, 24, 45]. The effect of winter warming comes with an early arrival of both spring and winter, extending the favorable period for mosquitoes to grow, develop and reproduce [42]. This physiological response involves both altered life histories and thermal adaptations to seasonality [46]. For this reason, the simulation of the warming effect cannot be treated homogeneously in any effort to evaluate or predict impacts on mosquitoes of different global species or genera [47]. The designed seasonal warming patterns become necessary to evidence how seasonal warming impedes or promotes *Ae. albopictus* growth in an annual development cycle. The short-term temperature variance as exemplified in the paper has a marked influence on the life cycle of epidemic vectors beyond the effects of long-term climate change [48–53].

By incorporating the 35-year climate dataset into the model, we have observed that the different degrees of all-year warming had very contrasting influences on the population dynamics. A modest temperature rise generally aided in the development with respect to the all-year accumulative population abundance (Fig. 3e); this effect was possibly caused by the elongated development time period the warming temperature induced (Fig. 3a, b). This finding further corroborates the fact that the projected planetary temperature rise of less than 2 °C in the years to come [54] will likely facilitate the mosquito growth in most world regions and promote its expansion to areas that are currently unfavorable. However, as the temperature continued to rise, the summer growth was inhibited. The intervening effect could be explained by laboratory controlled experiments that the preferable temperature range for *Ae. albopictus* adults to survive is 15–30 °C and the temperature of natural eradication is over 36 °C [55]. Thus, the summer temperature increase even to a small extent will exponentially raise the mortality rate and cause the population to plummet. This trend is also evidenced by research on the species in tropics and subtropics that the annual population dynamics has two peaks and a trough in summer [56, 57].

We have also observed that the different seasonal warming patterns mediated the development process very differently. The change of population abundance in all seasons but summer is possibly induced by the effect on diapause, a state of the mosquito eggs being dormant in winter in response to adverse environmental conditions [17, 58]. Typically, when the temperature drops below 9.5 °C, *Ae. albopictus* eggs transition into diapause and become completely inactive [59]. As the simulations illustrate, spring warming breaks the dormancy of diapause eggs and allows them to hatch earlier; autumn warming slightly extends the favorable period for reproduction and delays the diapause. However, these warming effects rendered cannot carry over to the following year. This result is very likely the effect of winter when the mosquito fully ceases from development and survives in terms of diapause eggs [17]. In addition, winter warming shortens the period of diapause. These effects are also evident in the SW2 and SW3 warming patterns, as shown in Additional file 1.

Admittedly, this study is flawed in several aspects. First, the design of the simulations assumes that the increased degree of temperature over the warming period is uniform. This assumption deviates from the uncertainties involved in global warming that the temperature rise is historically uneven on a daily basis and is documented with regional complications [23, 54]. To address the issue, future research on simulating the influence of warming should account for the natural fluctuating temperatures derived from historical climate data or projected by regional climate models [60]. Secondly, although both temperature and precipitation have been incorporated, the warming effects discussed in the paper only include temperature. In an atmospheric context, the change of the temperature gradient could contingently alter other climatic factors, including precipitation and humidity [2]. The constituent elements of global warming as predicted by scenarios in the Representative Concentration Pathways [61] and stochastic climatic conditions [62] should both be considered in the simulation as a way to improve the rigor of the model. Thirdly, an overlooked facet in the analysis is the evolutionary adaptation of the mosquito to climate change as a long-term shift in weather conditions [63]. The mechanistic link between population abundance and temperature is mediated by evolutionary responses that allow the mosquito to genetically evolve and facilitate its adaptation to warmer weathers and higher degrees of desiccation [64, 65]. Future research on evaluating and projecting the climatic suitability should take a precautionary approach to examining the yearly difference in the life cycle under similar climatic conditions.

## Conclusions

The rising presence and rapid expansion of dengue fever in the last few decades has raised considerable concerns about the nature and cause of the epidemic resurgence [66]. Although it is generally understood that global warming plays a potential role in increasing the incidence and geographical range of the disease [2], how climatic factors mechanistically alter the prevalence of the disease is a less elucidated territory with much complexity. In addition to human factors that increase vector-host interaction, the dengue transmission is dictated by the population dynamics of the vector *Ae.* *albopictus* mosquitoes. Our simulations with the 35-year climate data in a mathematical model show that the mosquito’s life cycle is sensitive to seasonal warming in different ways, where the development is inhibited by summer warming but is facilitated by both spring and winter warming. In this regard, the projected warming to a modest degree in future decades will most likely promote the growth and expansion of the species in high-latitude regions. Understanding this temperature-driven mechanism as one chain of the transmission events is critical to refining the thermal reaction norms of epidemic vectors under global warming as well as developing effective mosquito prevention and control strategies.

## Additional files

Additional file 1: Two-season (SW2) and three-season (SW3) warming patterns. (DOCX 16 kb)
Additional file 2: Regression analysis of the warming effects. (DOCX 14 kb)

## Abbreviations

DOY: Day of year
MPAD: Mechanistic population model of *Ae. albopictus* with diapause
SW1: Sinlge-season warming
SW2: Two-season warming
SW3: Three-season warming
YW: All-year warming
