# Supplementary figures and images for: How does the dengue vector mosquito Aedes albopictus respond to global warming?
Source: Parasit Vectors. 2017 Mar 11;10:140. doi: 10.1186/s13071-017-2071-2 (PMC5346228; doi:10.1186/s13071-017-2071-2)

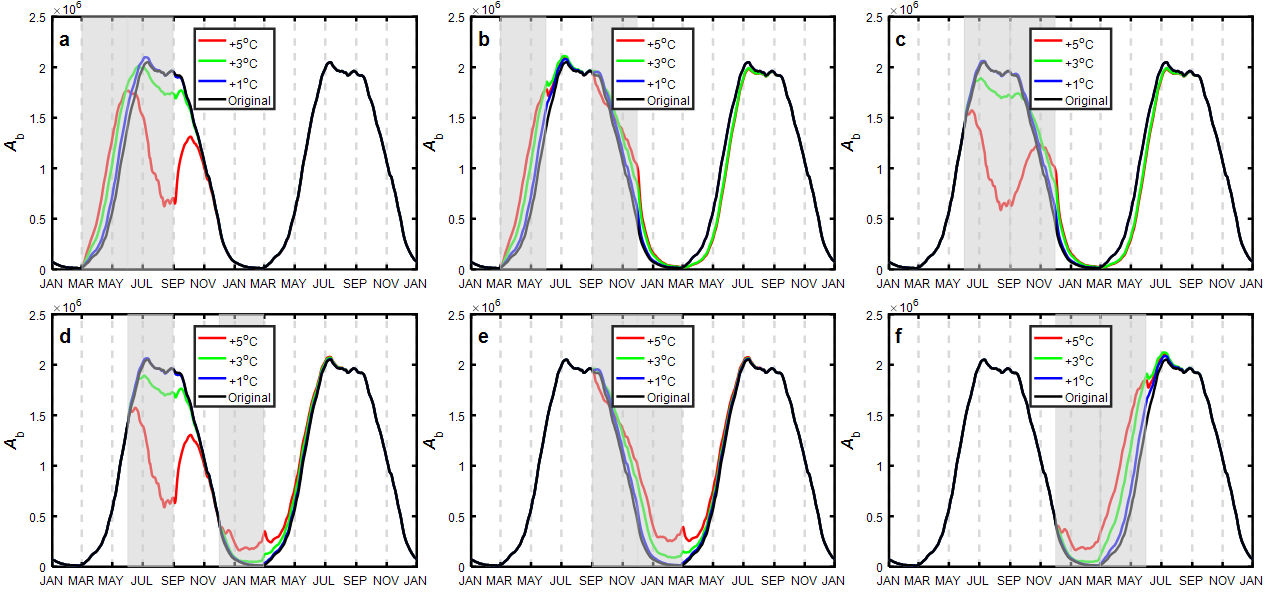

Supplement: Additional file 1: — Two-season (SW2) and three-season (SW3) warming patterns. (DOCX 16 kb) [file 13071_2017_2071_MOESM1_ESM.zip › Supporting Fig1.TIF]

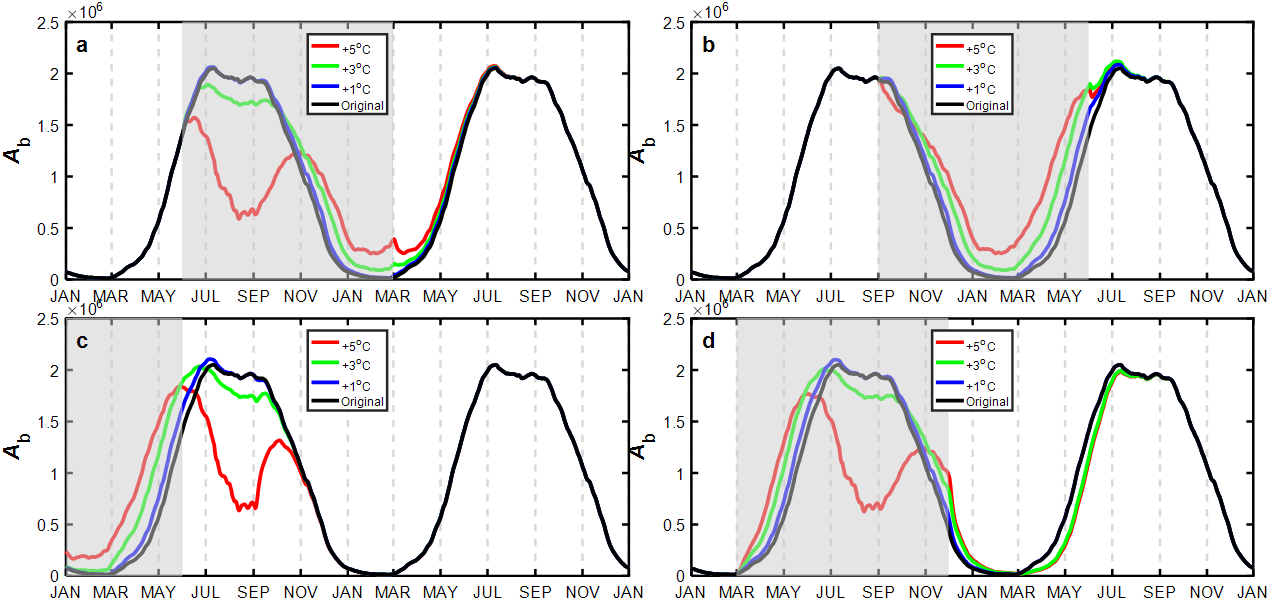

Supplement: Additional file 1: — Two-season (SW2) and three-season (SW3) warming patterns. (DOCX 16 kb) [file 13071_2017_2071_MOESM1_ESM.zip › Supporting Fig2.TIF]
