# Supplementary material for: How does the dengue vector mosquito Aedes albopictus respond to global warming?
Source: Parasit Vectors. 2017 Mar 11;10:140. doi: 10.1186/s13071-017-2071-2 (PMC5346228; doi:10.1186/s13071-017-2071-2)
Supplement: Additional file 2: — Regression analysis of the warming effects. (DOCX 14 kb) [file 13071_2017_2071_MOESM2_ESM.docx]

**Additional file 2.** Regression analysis of the warming effects

Supporting Tables 1 and 2 are regression equations established for the growth parameters of blood-fed adults as a function of the temperature increase (*ΔT*). The parameters include emergence time (*t*_e_), exit time (*t*_d_), peak time (*t*_p_), percentage change of peak value (*V*_peak_), and percentage change of population abundance (*V*_amount_). As can be seen from the *R*-square (R^2^), the correlations are evident, indicating that the population dynamics is relatively sensitive to the change of temperature.

**Supporting Table 1** Regression equations for *t*_e_, *t*_d_, and *t*_p_ as the dependent variable (*Y*) of *ΔT*

| Pattern | *t*_e_ | *t*_d_ | *t*_p_ |
| --- | --- | --- | --- |
| YW | *Y*^a^ = 99 - 8.4Δ*T* ^b^, *R*^2^ = 0.99 | *Y* = 349 + 5.0Δ*T*, *R*^2^ = 0.96 | *Y* = 198 - 8.3Δ*T*, *R*^2^ = 0.90 |
| SW1-Spr | *Y* = 96 - 4.9Δ*T*, *R*^2^ = 0.95 | *c* |  |
| SW1-Sum |  |  | *Y* = 196 - 5.5Δ*T*, *R*^2^ = 0.91 |
| SW1-Aut | **Y* ^d^ = 97 + 4.2Δ*T*, *R*^2^ = 0.83 | *Y* = 349 + 2.8Δ*T*, *R*^2^ = 0.90 |  |
| SW1-Win | **Y* = 96 + 3.9Δ*T* - 2.5Δ*T*^2^, *R*^2^ = 0.97 | *Y* = 349 - 1.7Δ*T* + 0.9Δ*T*^2^, *R*^2^ = 0.83 |  |
| SW2-Spr-Sum | *Y* = 96 - 4.9Δ*T*, *R*^2^ = 0.95 |  | *Y* = 197 - 8.1Δ*T*, *R*^2^ = 0.92 |
| SW2-Spr-Aut | *Y* = 96 - 4.9Δ*T*, *R*^2^ = 0.95  **Y* = 97 + 4.2Δ*T*, *R*^2^ = 0.83 | *Y* = 349 + 2.8Δ*T*, *R*^2^ = 0.90 |  |
| SW2-Sum-Aut | **Y* = 97 + 4.2Δ*T*, *R*^2^ = 0.83 | *Y* = 349 + 2.8Δ*T*, *R*^2^ = 0.90 | *Y* = 196 - 5.5Δ*T*, *R*^2^ = 0.91 |
| SW2-Sum-Win | **Y* = 96 + 4.1Δ*T* - 2.5Δ*T*^2^, *R*^2^ = 0.97 | *Y* = 350 - 1.7Δ*T* + 0.9Δ*T*^2^, *R*^2^ = 0.84 | *Y* = 196 - 5.5Δ*T*, *R*^2^ = 0.91 |
| SW2-Aut-Win | **Y* = 96 + 5.1Δ*T* - 3.1Δ*T*^2^, *R*^2^ =0.94 | *Y* = 344 + 9.0Δ*T*, *R*^2^ = 0.89 |  |
| SW2-Win-Spr | **Y* = 98 - 10.1Δ*T* + 0.2Δ*T*^2^, *R*^2^ = 0.99 | *Y* = 350 - 1.7Δ*T* + 0.9Δ*T*^2^, *R*^2^ = 0.83 |  |
| SW3-Sum-Aut-Win | **Y* = 96 + 5.1Δ*T* - 3.1Δ*T*^2^, *R*^2^ = 0.94 | *Y* = 344 + 9.0*ΔT*, *R*^2^ = 0.89 | *Y* = 196 - 5.5Δ*T*, *R*^2^ = 0.90 |
| SW3-Aut-Win-Spr | **Y* = 98 - 9.8Δ*T*, *R*^2^ = 0.99 | *Y* = 344 + 9.0Δ*T*, *R*^2^ = 0.89 |  |
| SW3-Win-Spr-Sum | *Y* = 97 - 8.9Δ*T*, *R*^2^ = 0.99 |  | *Y* = 198 - 8.7Δ*T*, *R*^2^ = 0.89 |
| SW3-Spr-Sum-Aut | *Y* = 96 - 4.9Δ*T*, *R*^2^ = 0.99  **Y* = 97 + 4.2Δ*T*, *R*^2^ = 0.84 | *Y* = 349 +2.8Δ*T*, *R*^2^ = 0.90 | *Y* = 197 - 8.1Δ*T*, *R*^2^ = 0.90 |

^a^*Y* is the variable for Year 2. All equations are significant at p-value < 0.001

^b^∆*T* is the temperature increase by 0.5–5 °C at an interval of 0.5 °C

^c^Blank slot means no significant correlation is identified

^d^**Y* is the variable for Year 3

**Supporting Table 2** Regression equations for *V*_peak_ and *V*_amount_ as the dependent variable (*Y*) of *ΔT*

| Pattern | *V*_peak_ | *V*_amount_ |
| --- | --- | --- |
| YW | *Y*^a^ = 0.0061 + 0.0250Δ*T* ^b^ - 0.0101Δ*T*^2^, *R*^2^ = 0.99 | *Y* = -0.0229 +0.1386Δ*T* -0.0266Δ*T*^2^, *R*^2^ = 0.91 |
| SW1-Spr | *Y* = -0.0004 + 0.0187Δ*T* -0.0029Δ*T*^2^, *R*^2^ = 0.99 | *Y* = -0.0023 + 0.0494Δ*T* - 0.0039Δ*T*^2^, *R*^2^ = 0.99 |
| SW1-Sum | *Y* = 0.0065 + 0.0082Δ*T* - 0.0116Δ*T*^2^, *R*^2^ = 0.99 | *Y* = -0.0151 + 0.0470Δ*T* - 0.0203Δ*T*^2^, *R*^2^ = 0.98 |
| SW1-Aut | **Y* ^c^ = 0.0027 - 0.0078Δ*T*, *R*^2^ = 0.87 | *Y* = 0.0125 + 0.0142Δ*T*, *R*^2^ = 0.86  **Y* = 0.0050 - 0.0181Δ*T*, *R*^2^ = 0.87 |
| SW1-Win | **Y* = 0.0002 + 0.0029Δ*T*, *R*^2^ = 0.99 | **Y* = -0.0069 + 0.0156Δ*T*, *R*^2^ = 0.96 |
| SW2-Spr-Sum | *Y* = -0.0048 + 0.0283Δ*T* - 0.0117Δ*T*^2^, *R*^2^ = 0.99 | *Y* = -0.0184 + 0.0982Δ*T* - 0.0240Δ*T*^2^, *R*^2^ = 0.93 |
| SW2-Spr-Aut | *Y* = -0.0004 + 0.0187Δ*T* - 0.0029Δ*T*^2^, *R*^2^ = 0.99  **Y* = 0.0027 - 0.0078Δ*T*, *R*^2^ = 0.87 | *Y* = -0.0051 + 0.0840Δ*T* - 0.0080Δ*T*^2^, *R*^2^ = 0.99  **Y* = 0.0050 - 0.0181Δ*T*, *R*^2^ = 0.85 |
| SW2-Sum-Aut | *Y* = 0.0065 + 0.0082Δ*T* - 0.0116Δ*T*^2^, *R*^2^ = 0.99 | *Y* = -0.0197 + 0.0869Δ*T* - 0.0265Δ*T*^2^, *R*^2^ = 0.97 |
|  | **Y* = 0.0029 - 0.0079Δ*T*, *R*^2^ = 0.88 | **Y* = 0.0051 - 0.0183Δ*T*, *R*^2^ = 0.86 |
| SW2-Sum-Aut | *Y* = 0.0065 + 0.0082Δ*T* - 0.0116Δ*T*^2^, *R*^2^ = 0.99 | *Y* = -0.0133 + 0.0453Δ*T* - 0.0193Δ*T*^2^, *R*^2^ = 0.98 |
|  | **Y* = 0.0002 + 0.0029Δ*T*, *R*^2^ = 0.99 | **Y* = -0.0067 + 0.0154Δ*T*, *R*^2^ = 0.96 |
| SW2-Aut-Win | **Y*= -0.0003 + 0.0025Δ*T*, *R*^2^ = 0.91 | *Y*= -0.0001 + 0.0293Δ*T* - 0.0011Δ*T*^2^, *R*^2^ = 0.99  **Y* = -0.0104 + 0.0160Δ*T*, *R*^2^ = 0.87 |
| SW2-Win-Spr | **Y* = -0.0003 + 0.0211Δ*T* - 0.0031Δ*T*^2^, *R*^2^ = 0.99 | **Y* = -0.0070 + 0.0452Δ*T*, *R*^2^ = 0.99 |
| SW3-Sum-Aut-Win | *Y* = 0.0065 + 0.0082Δ*T* - 0.0116Δ*T*^2^, *R*^2^ = 0.99 | *Y* = -0.0169 + 0.0818Δ*T* - 0.0236Δ*T*^2^, *R*^2^ = 0.97  **Y* = -0.0102 + 0.0159Δ*T*, *R*^2^ = 0.87 |
| SW3-Aut-Win-Spr | **Y* = 0.0001 + 0.0200Δ*T* - 0.0029Δ*T*^2^, *R*^2^ = 0.99 | *Y* = -0.0042 + 0.0238Δ*T*, *R*^2^ = 0.99  **Y* = -0.0039 + 0.0463Δ*T*, *R*^2^ = 0.99 |
| SW3-Win-Spr-Sum | *Y* = 0.0066 + 0.0260Δ*T* - 0.0099Δ*T*^2^, *R*^2^ = 0.98 | *Y* = -0.0179 + 0.1055Δ*T* - 0.0221Δ*T*^2^, *R*^2^ = 0.91 |
| SW3-Spr-Sum-Aut | *Y* = 0.0048 + 0.0283Δ*T* - 0.0117Δ*T*^2^, *R*^2^ = 0.99  **Y* = 0.0029 - 0.0079Δ*T*, *R*^2^ = 0.88 | *Y* = -0.0231 + 0.1383Δ*T* - 0.0302Δ*T*^2^, *R*^2^ = 0.93  **Y* = 0.0051 - 0.0183Δ*T*, *R*^2^ = 0.86 |

^a^*Y* is the variable for Year 2. All equations are significant at p-value < 0.001

^b^∆*T* is the temperature increase by 0.5–5 °C at an interval of 0.5 °C

^c^**Y* is the variable for Year 3
